# Mixing at the interface of the sneezing/coughing phenomena and its effect on viral loading

**DOI:** 10.1063/5.0073563

**Published:** 2021-11-19

**Authors:** Chandra Shekhar Pant, Sumit Kumar, Abhimanyu Gavasane

**Affiliations:** 1 Faculty of Mechanical Engineering, Technion-Israel Institute of Technology, Haifa, Israel; 2 Department of Mechanical Engineering, National Institute of Technology, Rourkela, India; 3 Department of Mechanical Engineering, B.M.S. College of Engineering, Bengaluru, India

## Abstract

The primary objective of this work is to investigate the mixing of droplets/aerosols, which originates from the sneezing/coughing (of possibly COVID-19 patient) with the ambient atmosphere. Effectively, we are studying the growth/decay of droplets/aerosols in the presence of inhomogeneous mixing, which focuses on the phenomena of entrainment of the (relatively) dry ambient air. We have varied the initial standard deviation, mean radius of the droplets/aerosols size distribution, and humidity of the ambient atmosphere to understand their effects on the final size spectra of droplets. Furthermore, a rigorous error analysis is carried out to understand the relative importance of these effects on the final spectra of droplets/aerosols. We find that these are vital parameters to determine the final spectra of droplets, which govern the broadening of the size spectra. Typically, broadening the size spectra of droplets/aerosols increases the probability of the virus-laden droplets/aerosols and thus could affect the transmission of infection in the ambient atmosphere.

## I. INTRODUCTION

The severity of COVID-19 has evolved from epidemic to pandemic. To date, 221 million people have been infected by COVID-19 and more than 4.58 million deaths have been claimed according to the latest online report published by the John Hopkins University (JHU).^1^ The worrying trend of the virus is its ability to mutate (Alpha, Beta, Gamma, and Delta)^2^ and evade the antibodies. To contain the spread of the virus, the government and non-governmental agencies across the globe have started an extensive vaccination program. In addition to that, the utility of mask, hand sanitizing, and social distancing is playing an important role to limit the spread of the pandemic.^3,4^ While focusing on the usage of mask and social distancing, important issues that need to be addressed are: (1) the virological loading of the droplets coming out through sneezing/coughing and (2) the effect of mixing of the coughing/sneezing and ambient air on the droplet size distribution (or the virological loading).

Previously, Dbouk and Drikakis^5^ studied the effect of ambient wind speed on the transmissibility of the virus-laden droplets/aerosols while coughing (herewith, we will use droplet and aerosol interchangeably while assuming both are the same). They argued that in the presence of wind, it is questionable that 2 m social distancing is sufficient to limit the extent of virus transmission. Furthermore, in their recent work of Dbouk and Drikakis^6^ they included various seasonal effects of the environment. They concluded that two pandemic peaks are possible because of the seasonal fluctuations in temperature, humidity, and wind speed. Similarly, researchers^7–20^ identified various environmental and/or social parameters to co-relate with the outbreak of COVID-19 virus. Bhardwaj and Agrawal^8^ found that the probability for the virus to stay in the ambient atmosphere increases with the increase in humidity content. In contrast, the works of Srivastava,^9^ Mecenas *et al.*^10^ demonstrated that a dry environment enhances the chances of transmissibility of the virus. From a modeling perspective, the sneezing/coughing action is modeled as a jet.^21,22^ Recently, Behera *et al.*^23^ using large eddy simulation modeled the human cough as a jet. They temporally investigated the presence of co-flow and highlighted the importance of co-flow in terms of distance traveled by the jet and the volume content. These features of the co-flow, in turn, help to give insight into the effect of ambient on the transmission rate. However, the presence of droplets could have made this study more realistic. Thus, the present work focuses on gaining more insight into the mixing phenomena while sneezing the droplets, including dispersion and evaporation/condensation. Perhaps, modeling the flow dynamics as the jet has been widely used in different engineering and research applications.^24–29^

The size distribution and number concentration of droplets ejected from humans vary in terms of biological actions (sneezing, coughing, talking, and easy respiration), age, and sex.^5,22,30–35^ It is to be noted that all the droplets ejected during different biological actions are not virus-laden. For instance, the works of Refs. 36–38 demonstrated that smaller droplets have higher viral loading. In contrast, the experimental work of Alonso *et al.*^39^ had shown that the viral loading is, in fact, higher in bigger droplets. Recently, Anand and Mayya^40^ have shown that for mild-moderate cases (viral load of less than 10^4^ RNA copies/ml), more than 99% of droplets below 60 *μ*m have a lesser tendency to carry the virus. Furthermore, they concluded that very fine droplets size of 2 *μ*m could be important for high severe cases. Thus, the behaviors of droplets size distribution during and after the mixing of the ambient air with the sneezing/coughing are essential to address the viral loading and transmissibility of the COVID-19 virus.

**FIG. 1.**
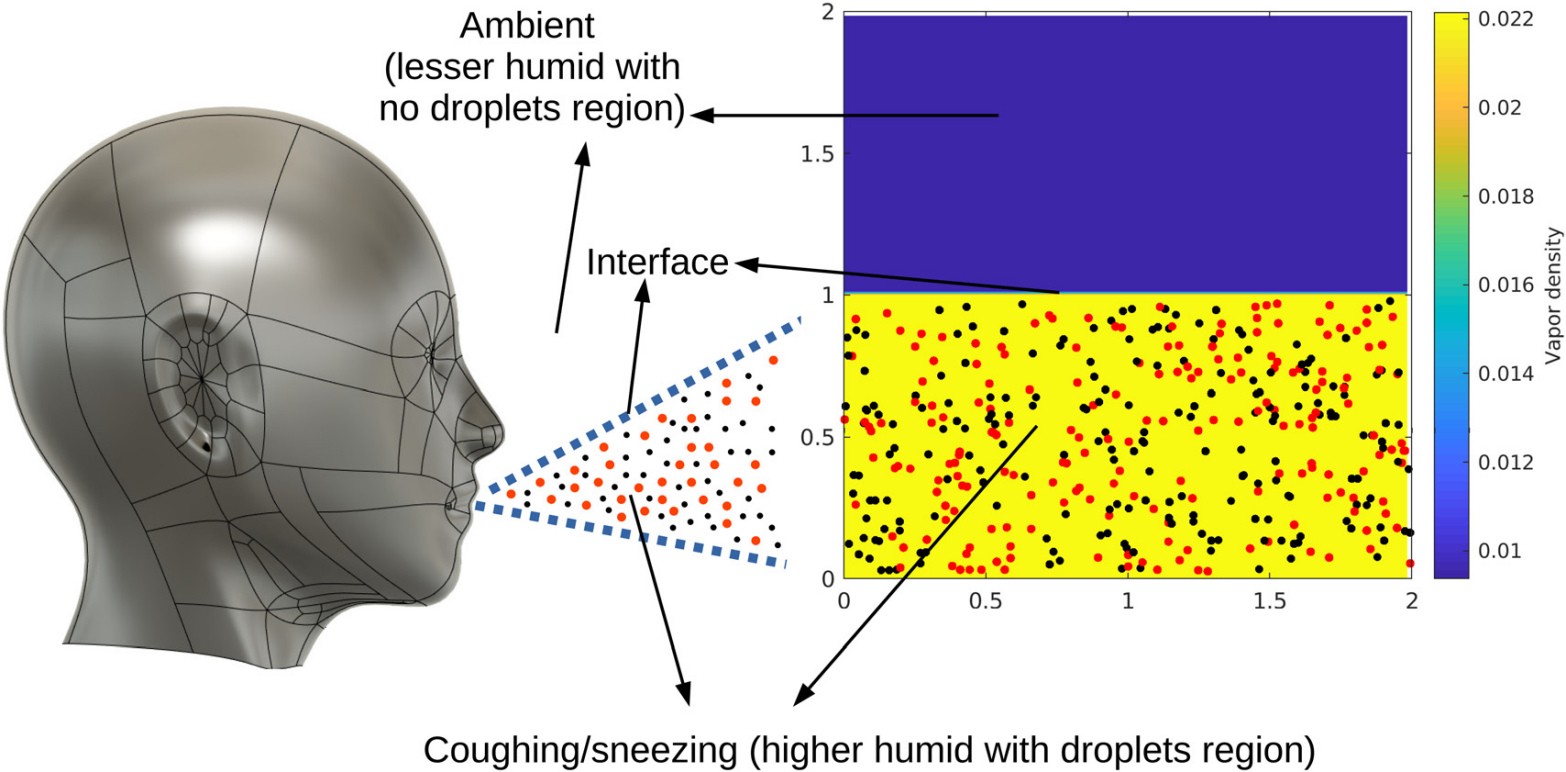
Schematic/illustration of the problem setup. The yellow-colored zone of the computational domain with droplets represent the coughing/sneezing zone, while the blue-colored zone represents the ambient atmosphere with no droplets. The coughing zone is saturated with the ambient atmosphere is sub-saturated; black and red dots represent the droplets, black dots signify the droplets with radius less than the mean radius, while red dots represent the larger droplets (bigger than the mean radius). The image file of the human face is taken from the online source, Reprinted with permission from See https://grabcad.com/library/human-face-4/ for “Library/human face” (last accessed September 20, 2021). Copyright 2021 Atharva Wagh.^44^

In the present work, we studied the mixing of the sneezing/coughing droplets with the ambient atmosphere. We considered a 2D periodic box, where the velocity, temperature, and vapor fields are solved using the Eulerian approach while the droplets are traced using the Lagrangian frame of reference. As an initial condition, the computational domain is divided into two halves. The bottom half consists of randomly spaced droplets placed in the supersaturated vapor field, while the upper half contains dry ambient air (shown in Fig. 1). Previously, a similar approach had been widely used in the cloud micro-physics community.^41–43^ We introduced a 2D synthetic turbulence velocity field having fixed turbulent kinetic energy, dissipation rate, and viscosity. This velocity field advects the droplets, temperature field, and vapor field. Thus we resolve the in-homogeneity in the vapor and temperature field with which each droplet interacts with the local density and temperature fields.

## II. PROBLEM SETUP, METHODOLOGY, AND GOVERNING EQUATIONS

The computational domain is initially segregated into two halves. The lower half consists of droplets implanted in a supersaturated vapor field, while the upper half comprises dry sub-saturated air without any droplets/virosols.^40^ Figure 1 shows that we are mimicking the mixing of coughing/sneezing action through the 2D computational domain. The coughing/sneezing zone contains higher humidity, while the ambient is a sub-saturated region. Moreover, we assume that only the sneezing/coughing has droplets, while there are no droplets in the sub-saturated zone. In the present work, using the scheme described in Sec. II B, we are able to impose $k^{-5/3}$ spectra; therefore, we are able to capture most of the features in the 3D version of this problem without incurring the high computational cost. 2D synthetic turbulence velocity field with fixed turbulent kinetic energy and dissipation rate is generated. This velocity field advects the droplets, temperature field, and vapor field, eventually mixing the species throughout the domain.

We perform numerical simulations of droplets evolving during turbulent mixing of dry and wet air parcels, in a 2D computational domain, of size $L_{x}\times L_{y}$. The motion of the fluid, vapor, and droplets is periodic in both the *x* and *y* directions. In these simulations of non-homogeneous turbulent mixing of droplets, we impose the motion of the fluid, and we resolve the advection of droplets, temperature, and vapor to make sure that the droplets interact only with the vapor field in their corresponding/immediate vicinity [Fig. 4(b)]. We implicitly assume that the pressure and volume of the 2D parcel is invariable with time. Below, we describe the equations that are used to evolve the droplets (Sec. II A) and the field variables (Sec. II B).

### A. Governing equations for evolution of droplets

We used the Lagrangian approach to evolve the droplets, such that the position of every droplet is resolved, and the size and temperature of each droplet are explicitly evolved separately. The droplets' growth rate is given by^45,46^

$$\frac{dR_{i}^{2}}{dt}=-\frac{\left(\rho_{v,s}(t)-\rho_{v}(x^{i})\right)}{\rho_{w}}D_{v1},$$ (1)

where $R_{i}(t)$ is the radius of the ith droplet at time *t*, and $\rho_{v}(x^{i})$ is the vapor density field at the location of the droplet, $x^{i}$. Here, $D_{v1}$ is defined^47^ in terms of the temperature of the vapor $T_{a}(x^{i},t)$ at the droplet location,

$$D_{v1}=\frac{D_{0}}{1.0+D_{0}L^{2}\rho_{v,s}^{i}/(k_{a}R_{v}T_{a}{\left(x^{i},t\right)}^{2})},$$ (2)

where *D*_0_ is the diffusivity of the vapor, *k_a_* is conductivity of water vapor, and $D_{v1}$ is defined^47^ in terms of the temperature of the vapor $T_{a}(x^{i},t)$ at the droplet location. Also, $\rho_{v,s}^{i}(t)$ is the saturation vapor pressure at the droplet,

$$\rho_{v,s}^{i}(t)=\frac{611}{R_{v}T_{a}(x^{i},t)}\text{exp}\;\left[\frac{L}{R_{v}}\left(\frac{1}{273}-\frac{1}{T_{a}(x^{i},t)}\right)\right],$$ (3)

here *L* is waters' latent heat of condensation, and *R_v_* is the gas constant for vapor. In turn, the droplets' temperature changes because of the latent heat release due to condensation, also by the conductive heat interaction from the surrounding air.

$$\frac{dx^{i}}{dt}=u(x^{i}),$$ (4)

where u(x,t) is the fluid velocity. The sedimentation and droplet inertia is ignored, while assuming that the Stokes number is very small ( ≪1) for the droplets. We also assume, following following Ref. 47, that the temperature of droplet is equal to the local domain temperature. The values of $T_{a}(x^{i},t),\rho_{v}(x^{i},t)$, and $u(x^{i},t)$ are calculated using linear interpolation of $T_{a}(x,t),\;\rho_{v}(x,t)$, and u(x,t), respectively, onto the droplet location $x^{i}$.

### B. Governing equations for field variables evolution

The description of the governing equations to evolve the field variables viz. velocity, temperature, vapor density are explained in this section. Using the synthetic turbulence model, given by Refs. 48 and 49 unsteady velocity field u(x,t) is generated. The velocity field is discretized in terms of *N_x_* and *N_y_* Fourier modes in the *x* and *y* directions, and each Fourier mode is evolved using a stochastic Langevin equation. An isotropic, statistically stationery, divergence- free, homogeneous stochastic, and divergence-free two-dimensional velocity field having Karman–Obukhov energy spectra is generated via this method. The energy spectra *E*(*k*) obtained from the synthetic velocity field is compared against the analytical expression of Ref. 48 given by

$$E(k)=\frac{\epsilon }{4\pi \tau }k^{3}{\left(1+\lambda^{2}k^{2}\right)}^{-7/6},$$ (5)

here, *k* is wavenumber, *ϵ* is the dissipation rate, *τ* is the timescale, and *λ* is the size of the most energetic scales in the flow. During growth/condensation and/or evaporation, each droplet generates/liberates vapor mass, given by the following source terms:

$$S_{M}^{i}=2\pi R_{i}D_{v1}(\rho_{v,s}^{i}-\rho_{v}(x_{i})).$$ (6)

Considering this [Eq. (6)] source terms, the conservation of vapor density and energy in the ambient air can be written as

$$\frac{\partial \rho_{v}}{\partial t}+u\cdot \nabla \rho_{v}=-\sum_{i=1}^{N_{s}}S_{M}^{i}n_{d}\delta^{3}(x-x_{i})+D_{v}\nabla^{2}\rho_{v}+\nu_{t}\nabla^{2}\rho_{v},$$ (7)

$$\frac{\partial T_{a}}{\partial t}+u\cdot \nabla T_{a}=-\frac{1}{C_{a}\rho }\sum_{i=1}^{N_{s}}n_{d}\left[LS_{M}^{i}\right]\delta^{3}(x-x_{i})+\frac{k_{a}}{\rho C_{a}}\nabla^{2}T_{a}+\nu_{t}\nabla^{2}T_{a}.$$ (8)

Here, *C_a_* is the specific heat capacity of air, *ρ* is the density of the air, and δ(x) is the Dirac delta function. *S_M_* or the source terms for the mass contributes to the energy equation because of the contribution from the latent heat *L*. *ν_t_* is the artificial viscosity obtained using $\nu_{t}=0.5\frac{u_{\mathrm{rms}}^{2}\tau }{{\left(\Delta x/\lambda \right)}^{0.66}}$. This expression for *ν_t_* is obtained assuming that for a given length scale *l*, the velocity scale is *u_l_* which scales like $u_{l}\propto l^{1/3}$. We also note that eddies of size *λ* have a velocity scale *u_rms_*. For sub-grid scale turbulence, which have a size Δx, velocity scale is then given by $u_{\Delta x}∼u_{\mathrm{rms}}{\left(\Delta x/\lambda \right)}^{1/3}$. The timescale for eddies of all scales in the synthetic turbulence is *τ*. The net diffusion coefficient for the sub-grid scales is therefore proportional to $u_{\Delta x}^{2}\tau ∼\frac{u_{\mathrm{rms}}^{2}\tau }{{\left(\Delta x/\lambda \right)}^{0.66}}$. The factor of 0.5 was determined via trial and error by ensuring that the dispersion of passive scalars by the turbulent field stayed the same over several different grid resolutions. We used a standard finite-difference method to evolve Eqs. (7) and (8).

## III. INITIAL CONDITIONS AND/OR PARAMETERS

The computational domain is periodic in *x* and *y* direction. The computation domain is set as $L_{x}=L_{y}=2$ m in order to replicate the domain of influence of sneezing/coughing region.^50–52^ While our simulations are 2D, we provide computational box depth *L_z_* equal to the average inter-droplet spacing, i.e., we assign $L_{z}=\sqrt{L_{x}L_{y}/N}$, where *N* is the total number of droplets. This ensures the same average inter-droplet spacing in all three directions. The effective droplet number concentration is then equal to $N/(L_{x}L_{y}L_{z})={\left(N/(L_{x}L_{y})\right)}^{3/2}$. The number density of droplets is fixed at 2828 cm^−3^. The fluid timescale is controlled by the relation given by

$$\tau_{f}=\frac{\lambda^{2/3}}{\epsilon^{1/3}},$$ (9)

where *λ* is the length scale and *ϵ* is the dissipation rate. We fixed the $\lambda =L_{x}/5$ and ϵ=0.01 $m^{2}s^{-3}$. This value of *ϵ* corresponds to the atmospheric conditions.^53^ The Gaussian distribution is used for the initial droplet size spectra. We assume that the droplet radius range of 2–3 *μ*m is completely evaporated and follows a lognormal distribution. Further details of the initial parameters used in the simulations along with the references are detailed in Table I.

**TABLE I.** Parameters used in the numerical simulations.

Sr no.	Parameter	Present work	Range (References)
1	Length of domain (*L_x_* = *L_y_*)	2 m	2 m^[^^50–52^^]^
2	Dissipation rate (*ϵ*)	0.01 $m^{2}s^{-3}$	$10^{-5}$– $10^{-2}$ $m^{2}s^{-3}$^[^^53^^]^
3	Relative humidity (*RH*)	0.4–0.9	0.1–0.9^[^^8,22^^]^
4	Droplets range (*R_min_*, *R_max_*)	20–100 *μ*m	2–1000 *μ*m^[^^22,54^^]^
5	Cut of radius (*R_cut_*)	2 *μ*m	2 *μ*m^[^^40^^]^
6	Number density of droplets (*n_d_*)	2828 cm^–3^	1– $2.5\times 10^{3}$ cm^−3^^[^^30,40,55^^]^

## IV. VALIDATION

We could not compare directly the present work with the previous literature, since there is no exact/similar work done in the past. Thus the present solver is validated separately for droplet growth or homogeneous condensational model (also called the parcel model) and for the fields/velocity evolution.

### A. Validation for the homogeneous condensational model

In Sec. II A, complete convective diffusive equations are explained while for validating the droplets growth model, we considered the volume averaged (or homogeneous) fields. To avoid confusion, we are re-writing the complete set of equations for droplet growth (also, previously explained in Sec. II A).

The droplet growth rate is governed by^45,46^

$$\frac{dR_{i}^{2}}{dt}=-\frac{(\rho_{v,s}(t)-\left\langle \rho_{v}\right\rangle )D_{v1}}{\rho_{w}},$$ (10)

where $R_{i}(t)$ is the radius of the *i*th droplet at time *t*, *ρ_w_* is the density of water, and $\left\langle \rho_{v}\right\rangle (t)$ is the vapor density field. The ensemble averaging operator ⟨·⟩ essentially implies an averaging over space here. *D*_0_ is the diffusivity of water vapor and *k_a_* is conductivity of water vapor. The effective diffusivity $D_{v1}$^47^ is given as

$$D_{v1}=\frac{D_{0}}{1.0+D_{0}L^{2}\rho_{s,v}(t)/(k_{a}R_{v}{\left\langle T_{a}\right\rangle }^{2})},$$ (11)

$\rho_{v,s}(t)$ is the saturation vapor pressure corresponding to the domain temperature, $\left\langle T_{a}(t)\right\rangle$ (in Kelvin)

$$\rho_{v,s}=\frac{611}{R_{v}\left\langle T_{a}(t)\right\rangle }\text{exp}\;\left[\frac{L}{R_{v}}\left(\frac{1}{273}-\frac{1}{\left\langle T_{a}\right\rangle (t)}\right)\right],$$ (12)

here *L* is the latent heat of condensation for water, and *R_v_* is the vapor gas constant. The expression for $D_{v1}$ [Eq. (11)] can be derived by using the fact that the diffusion timescale of the vapor and temperature is small, and that the temperature difference between the droplet and its surroundings is negligible;^47^ this approximation eliminates the requirement of evolving the temperature of each droplet. In general, the homogeneous air parcel will be having a vertical velocity, and the atmosphere will have a vertical temperature gradient (or lapse rate). The equations for the temperature and vapor in the parcel are therefore given by^46^

$$\frac{d\left\langle T_{a}\right\rangle }{dt}=-\frac{1}{C_{a}\rho V}\sum_{i}\left[LS_{M}^{i}\right]-W\Gamma ,$$ (13)

$$\frac{d\left\langle \rho_{v}\right\rangle }{dt}=-\frac{1}{V_{a}}\sum_{i}S_{M}^{i},$$ (14)

where *W* is the updraft velocity, Γ is the lapse rate ( $\Gamma =g/C_{a}$), *g* is acceleration due to gravity, *C_a_* is the specific heat capacity of air, *ρ* is air density, and *V_a_* is the volume of the air parcel. To be noted that the lapse rate term is specifically included to validate this model, in the rest of the manuscript, this term is neglected. The mass source term $S_{M}^{i}$ given by [similar to that explained in Sec. II B, Eq. (6)]

$$S_{M}^{i}=2\pi R_{i}D_{v1}(\rho_{v,s}(t)-\left\langle \rho_{v}\right\rangle ).$$ (15)

The above expression follows directly from Eq. (10), since $S_{M}^{i}=-\rho_{w}d((4/3)\dot{\pi }R_{i}^{3})/dt$. Standard Runge–Kutta second order time stepping is performed to evaluate Eqs. (10), (13), and (14). Time stepping for both the bulk parcel parameters and droplets is $10^{-2}$ s.

**FIG. 2.**
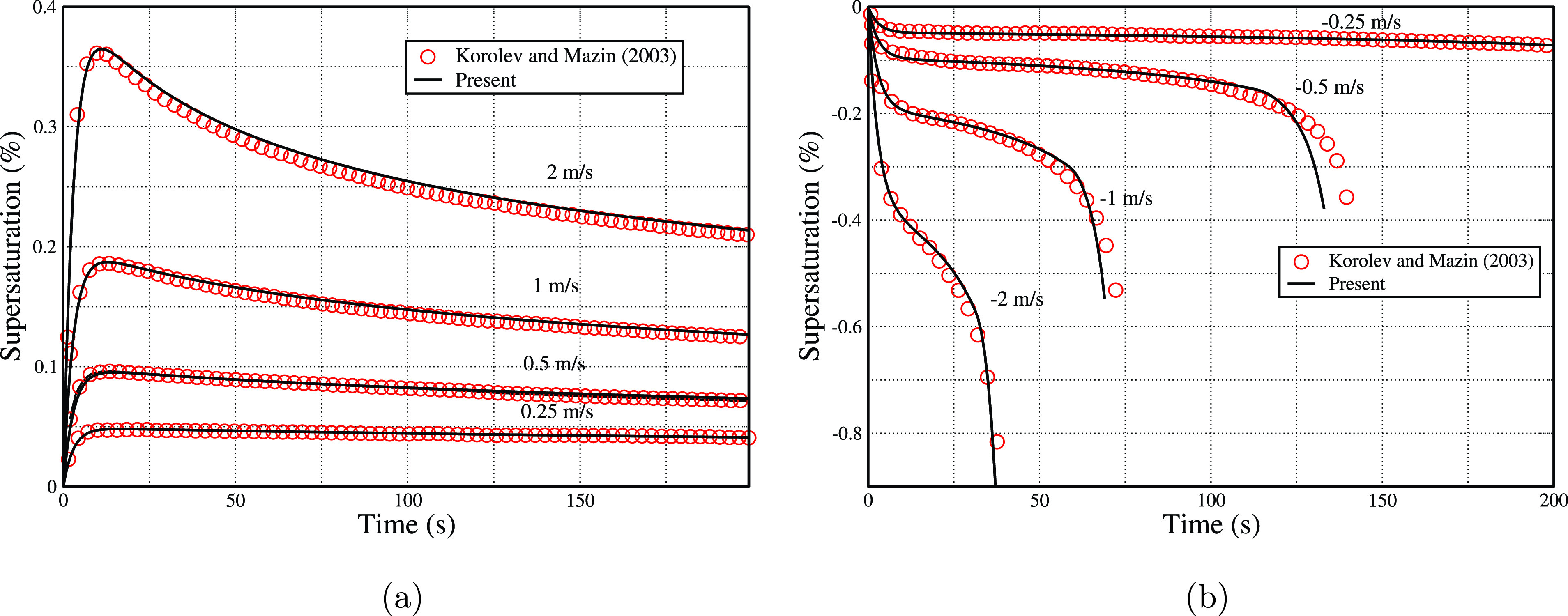
Time history of supersaturation for different (a) updraft velocities and (b) downdraft velocities, for present work and the Korolev and Mazin.^56^

The present numerical simulations are validated against the analytical/numerical model proposed by Korolev and Mazin.^56^ The mono-dispersed droplets of mean radius 5 *μ* m with an initial number concentration of 200 ${\text{cm}}^{-3}$ are placed in a parcel with saturated conditions (i.e., supersaturation of 0), at temperature of 0 °C. Figures 2(a) and 2(b) show the variation of supersaturation ( $S=(\left\langle \rho_{v}\right\rangle /\rho_{v,s}-1)$) against time with the different updraft and downdraft velocities, respectively. In Fig. 2(a) initially the supersaturation increases with time because of the decrease in temperature, which leads to the decrease in the saturated pressure (according to the Clausius–Clapeyron relationship). Simultaneously, the vapor inside the parcel also starts condensing on the droplets, which in turn releases latent heat into the parcel and reduces the supersaturation. With a higher updraft velocity, the maxima in supersaturation are higher and in turn lead to a larger time for supersaturation to attain the quasi-static value. Similar argument is valid for the cases with downdraft. For different updraft and downdraft velocities, the results show that the present simulations [Figs. 2(a) and 2(b)] are in good agreement with the model proposed by Korolev and Mazin.^56^

### B. Validation for field/velocity evolution

**FIG. 3.**
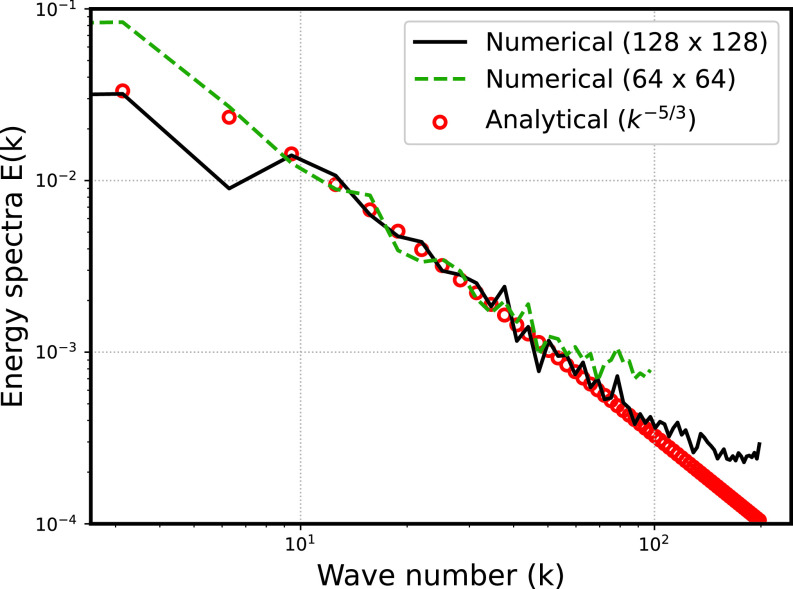
Grid independence and validation of the velocity field.

Figure 3 shows the validation of energy spectra corresponding to different resolutions. The resolved part of the energy spectra does not appear to change significantly for the different grid sizes and appear to agree reasonably well with the analytical expression for the spectra. To avoid truncation of the energy spectra (i.e., to ensure that the $k^{-5/3}$ part of energy spectra does not disappear), we used $N_{x}\times N_{y}=128\times 128$ in our present manuscript.

## V. RESULTS AND DISCUSSION

**FIG. 4.**
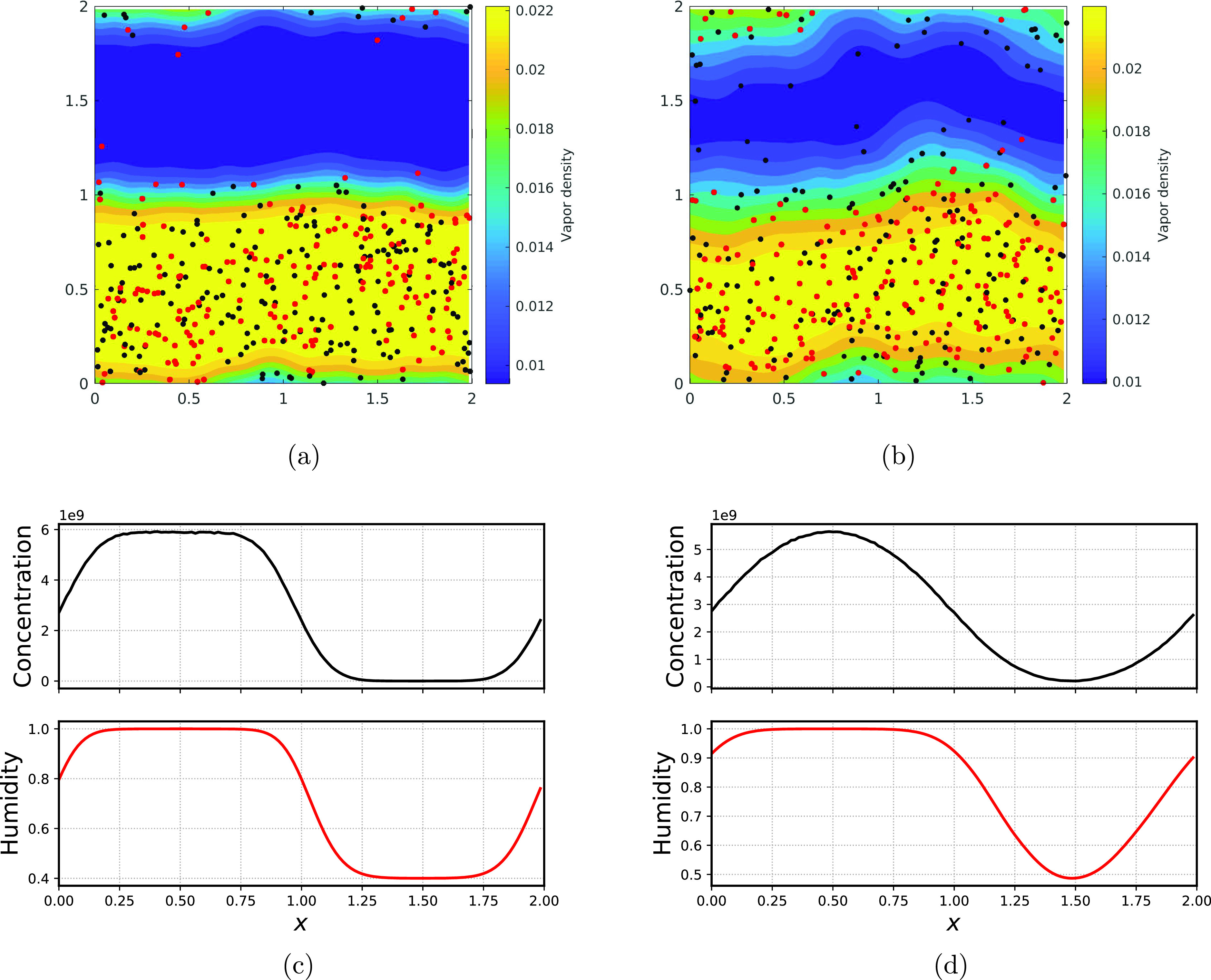
(a) Snapshot of mixing at *t *=* *1 s and (b) *t *=* *4 s (c) subplot of concentration and humidity at *t *=* *1 s and (d) *t *=* *4 s.

We assume that the droplets and surroundings are at the same room temperature of 298 K in the rest of the manuscript. The humidity contrast triggered by the stochastic velocity initiates the mixing at the interface and thereby propagates in the complete computational box. Figures 4(a) and 4(b) show the quantitative snapshots at different time instant of the mixing. Initially, at the interface (between the sneezing and ambient air), the droplets tend to evaporate because of the lesser vapor content, while the droplets that are still in the saturated region tend to grow because of the condensation. The black color dots signify the smaller (having a radius less than the mean radius of the domain) droplets, and red color dots imply bigger (radius bigger than the mean radius) droplets. Over time, the gradient in vapor density and droplet concentration smooths and finally homogenizes the computational domain. Figures 4(c) and 4(d) show the corresponding quantitative snapshots of variation of humidity and concentration of droplets. This is the common mixing characteristics of the droplets and vapor density; however, in the next sections, we will seek the effects of changing the initial conditions of the computational domain and/or droplets in the final spectra of droplets, which eventually govern the transmissibility of the infection through droplets.

### A. Effect of standard deviation of droplet size spectra

**FIG. 5.**
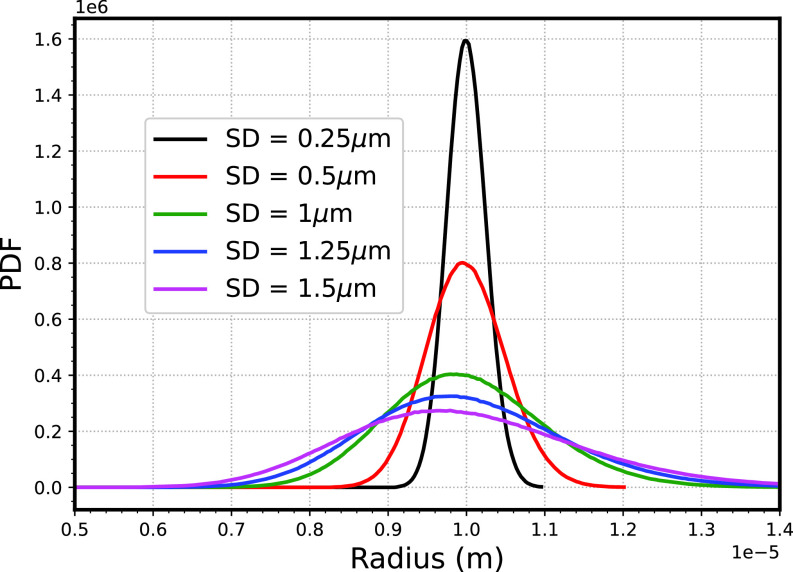
Initial size spectra of droplets corresponding to varying initial standard deviation.

**FIG. 6.**
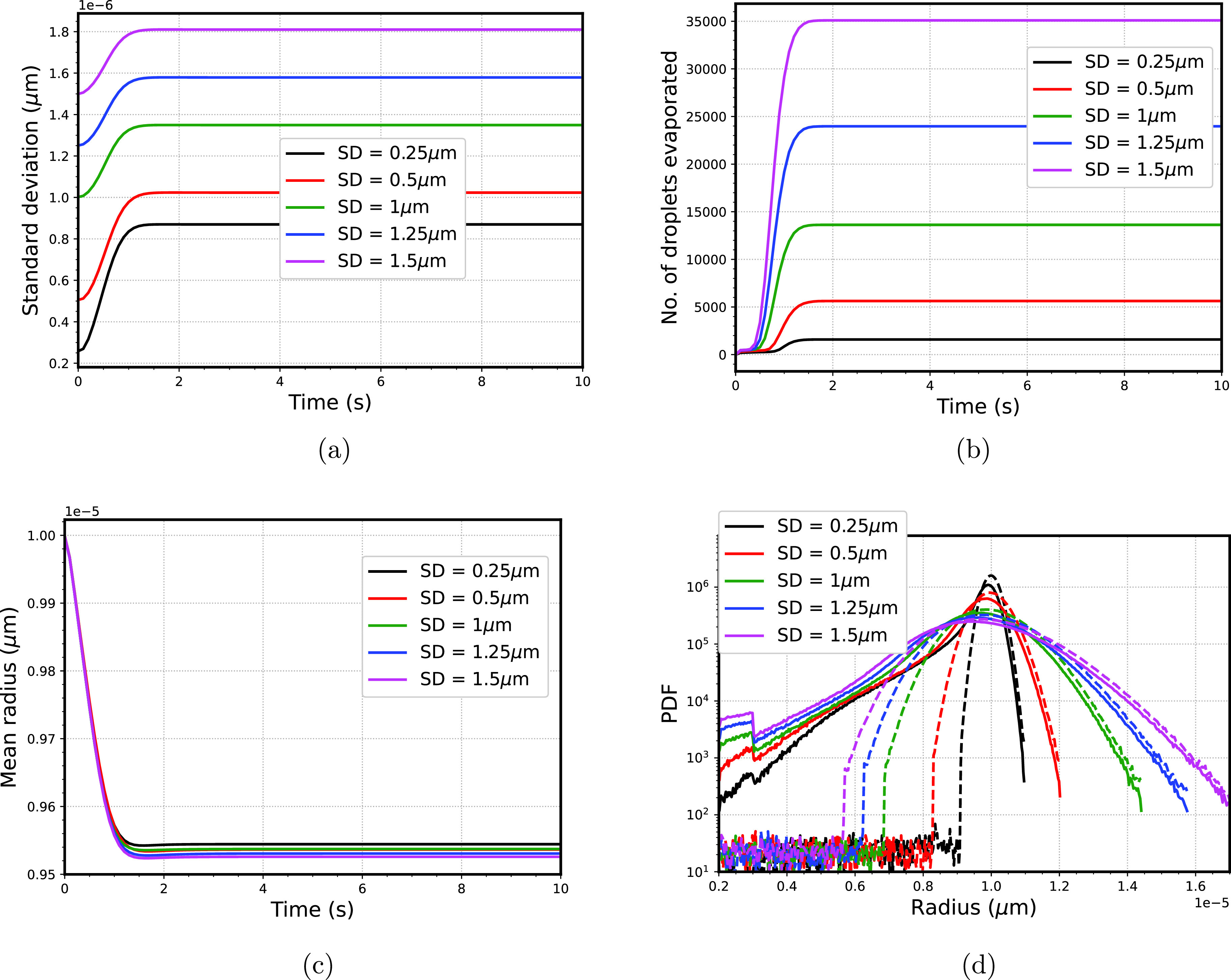
(a) Time variation of standard deviation (b) variation of number of droplets evaporated with time (c) mean radius variation of droplet size spectra with time (d) final droplet size spectra of droplets, and the dashed line shows the initial spectra corresponding to different standard deviation.

We considered five different standard deviations of droplet size distribution ranging from narrow (0.25 *μ*m) to broad (1.5 *μ*m), while maintaining the same mean radius of 10 *μ*m, shown in Fig. 5 with humidity at the dry section *RH *=* *0.4 and at wet region *RH *=* *1. Specifically, we took standard deviation of 0.25, 0.5, 1, 1.25, and 1.5 *μ*m. Figures 6(a)–6(c) show that time variation of standard deviation, number of droplets evaporated, and mean radius cease to change after time > 1.5 s since, by this time, the gradients in the computational domain are smoothened out. Figure 6(a) shows that broader droplet size spectra end up with a larger standard deviation (as compared to the lower standard deviation); however, the slope or the relative increase in the standard deviation is the smallest for this case. Further discussion on this issue will be explained in Sec. V D. Meanwhile, because of the presence of a higher number of smaller droplets for broad spectra of droplets as compared with the narrow spectra of droplets, the number of droplets that are evaporated (range of 2–3 *μ*m) is higher [shown in Fig. 6(b)]. However, the complete evaporation of the smaller droplets has a marginal effect on the overall mean radius of the droplets; thus, the decrease in mean radius is least for the broad size spectra [shown in Fig. 6(c)]. Finally, at time =10 s, the final droplet size spectra are shown in Fig. 6(d).

Anand and Mayya^40^ showed that with the increase in the severity of the cases (from moderate to severe), the value of average RNA copies/mL also increases from 10^2^ to 10^6^. With the increase in average RNA copies/mL values, the cutoff (lesser probability of virus) average mean diameter of droplets also decreases. For instance, for the case of moderate infection, the cutoff is around 60 *μ*m, while for the severe case, the cutoff could be decided close to 15 *μ*m. It is noted that these are the ranges for 0.1% virus-containing droplet having 10^4^ RNA copies/ml and 10^6^ RNA copies/ml for moderate and severe cases, respectively. Thus, if the initial distribution ejected during sneezing/coughing is broader (in severe cases) then after mixing with the ambient atmosphere, this could further broaden the droplet size spectra and thus finally making the scenario more hazardous.

### B. Effect of variation of mean radius of droplet size spectra

**FIG. 7.**
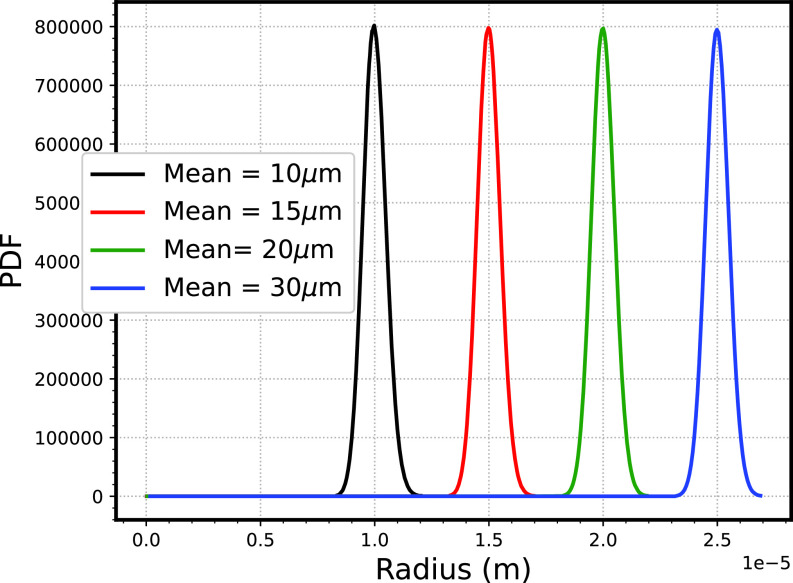
Initial size spectra of droplets corresponding to varying initial mean radius.

**FIG. 8.**
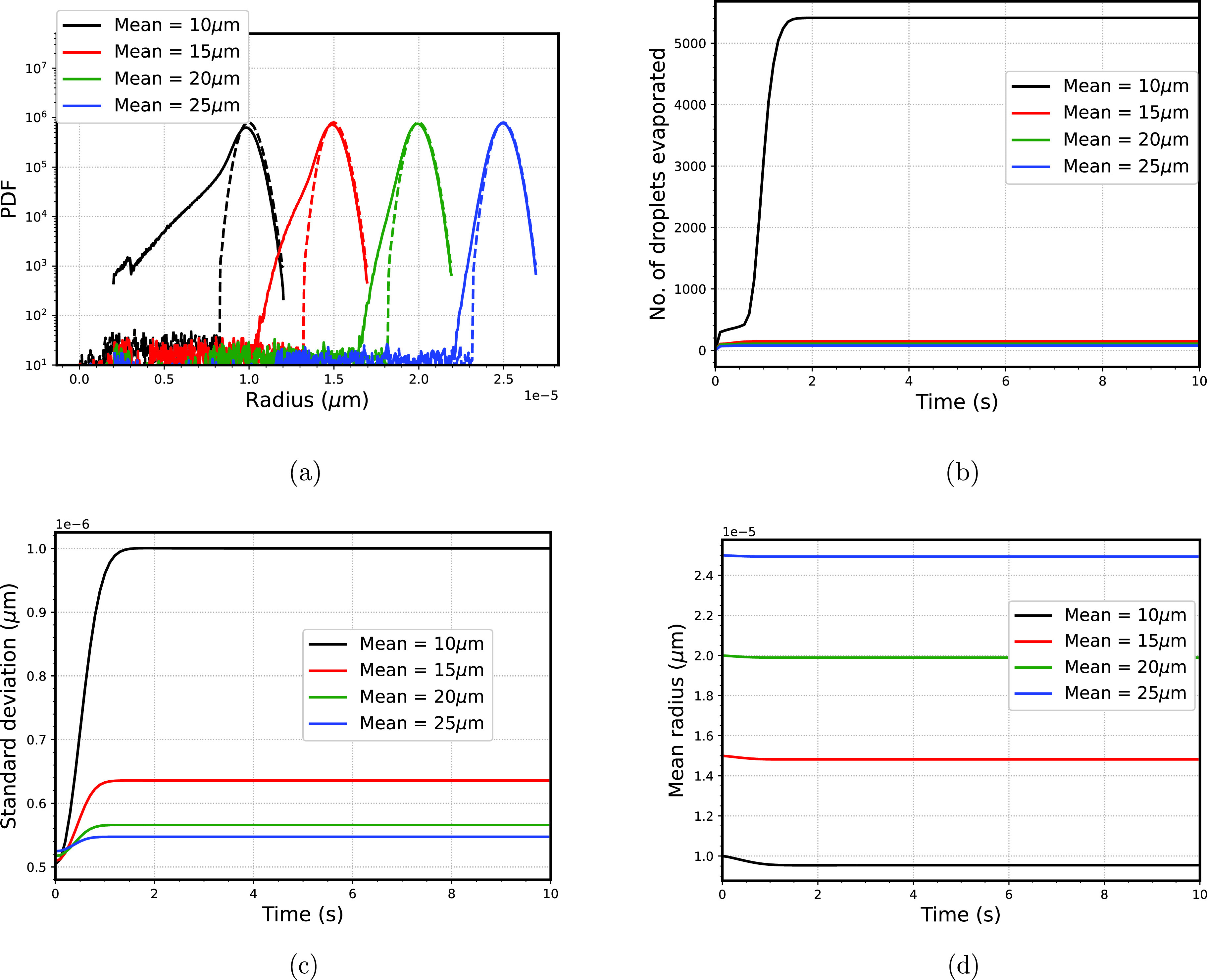
(a) Final droplet size spectra of droplets, dashed line shows the initial spectra corresponding to different standard deviation (b) variation of number of droplets evaporated with time (c) time variation of standard deviation (d) mean radius variation of droplet size spectra with time.

We varied the mean radius of the droplet size spectra as 10, 15, 20, and 30 *μ*m while maintaining the same standard deviation of 0.5 *μ*m (shown in Fig. 7). Figure 8(a) shows the final droplet size spectra for different mean radii. For the minimum mean radius of 10 *μ*m, the broadening of the droplet distribution is observed. This is because, in the case of the smaller mean radius of droplets, the smaller droplets tend to evaporate completely (range of 2−3 *μ*m), thereby saturating the computational domain. As seen in Fig. 8(b), the highest number of evaporated droplets are for the case of minimum radius of 10 *μ*m. In contrast, the smallest droplets that are evaporated correspond to the highest mean radius of droplet spectra (25 *μ*m). Furthermore, this broadening of the droplet size spectra is quantified in Fig. 8(c) by plotting the variation of standard deviation with time. The standard deviation increases twice for the lowest mean radius case (10 *μ*m). In the case of a larger mean radius, the partial evaporation of the larger droplets prohibits the rest of the smaller droplets from evaporating completely; therefore, there is an insignificant variation of mean radius for the larger mean radius (25 *μ*m). In terms of transmission, the presence of bigger droplets (or larger mean radius) prohibits the broadening of droplet size spectra; thus, the probability of spreading the virus through severe cases decreases.

### C. Effect of variation of environmental humidity

**FIG. 9.**
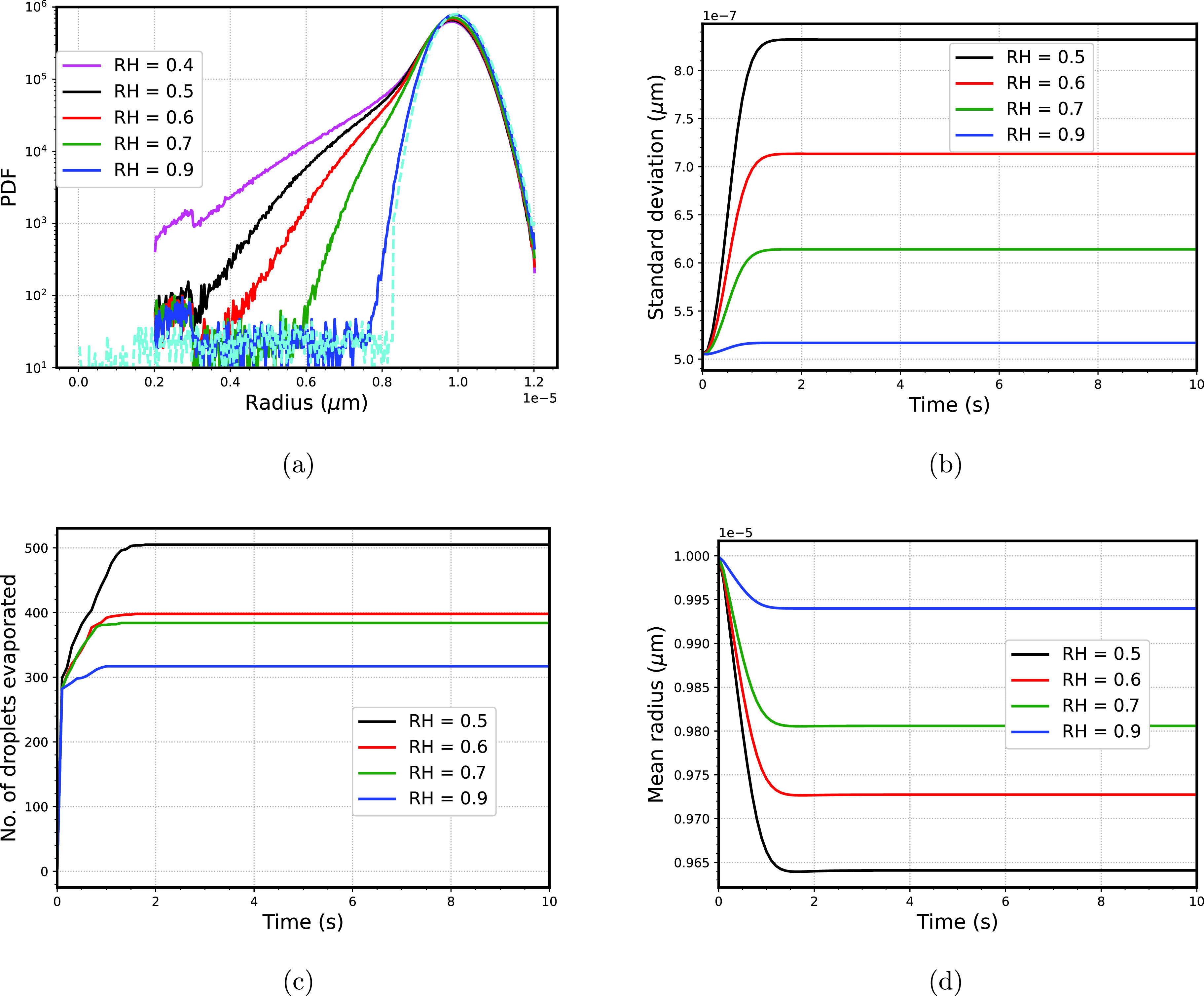
(a) Final droplet size spectra of droplets, dashed (cyan) line shows the initial spectra (b) variation of number of droplets evaporated with time (c) variation of number of droplets evaporated with time (d) mean radius variation of droplet size spectra with time.

The humidity of the ambient environment varies from region to region; thus to consider this effect, we varied the relative humidity (*RH*) of the dry section as 0.4, 0.5, 0.6, 0.7, and 0.9 (the results obtained corresponding to *RH *=* *0.4 are not explained, since these are already discussed in Sec. V B). The standard deviation and mean radius of the droplet size spectra are fixed at 0.5 and 10 *μ*m, respectively. With the increase in the dryness at the sub-saturated section of the computational domain, the droplet size spectra broaden. Figure 9(a) shows the effect of *RH* of the dry section, increasing the *RH* of the sub-saturated region restricts the evaporation of the droplets leading to the decrease in the standard deviation of droplet size spectra. Figure 9(b) shows that with the increase in the *RH* value in the dry section, the standard deviation of droplet size spectra decreases. This effect could be understood as the implication of the droplets evaporated for the different *RH* values. Figure 9(c) shows that because of insufficient humidity in the computational domain for the minimal *RH* case, the number of droplets that undergoes evaporation is the highest, and this number decreases with the increase in the humidity level. As expected, with the higher number of droplets evaporation, the mean radius of the droplet size spectra decreases [shown in Fig. 9(d)]. The higher humidity contrast between the sneezing zone and the ambient atmosphere leads to broadening the droplet size spectra. Previously, as has been reported by Bhardwaj and Agrawal^8^ that chances of transmission increase fivefold because of the humid environment. Instead, we found that, that because of higher humidity contrast, the broadening of the droplet size spectra leads to the formation of smaller droplets which, in the case of severe infections, have a higher probability of carrying virus (provided the temperature of ambient and sneezing zone is assumed to be constant). This could be interpreted as a scenario of higher virus density in the droplet. In the process of evaporation, even if we assume that only the liquid-vapor evaporates while the virus content remains intact in the droplet. Then in terms of viral density, the probability of the transmission is higher. These finds related to humidity are in line with the work of Mecenas *et al.,*^10^ who concluded that high humidity and high temperature reduce the transmission of the virus.

### D. Error analysis

**FIG. 10.**
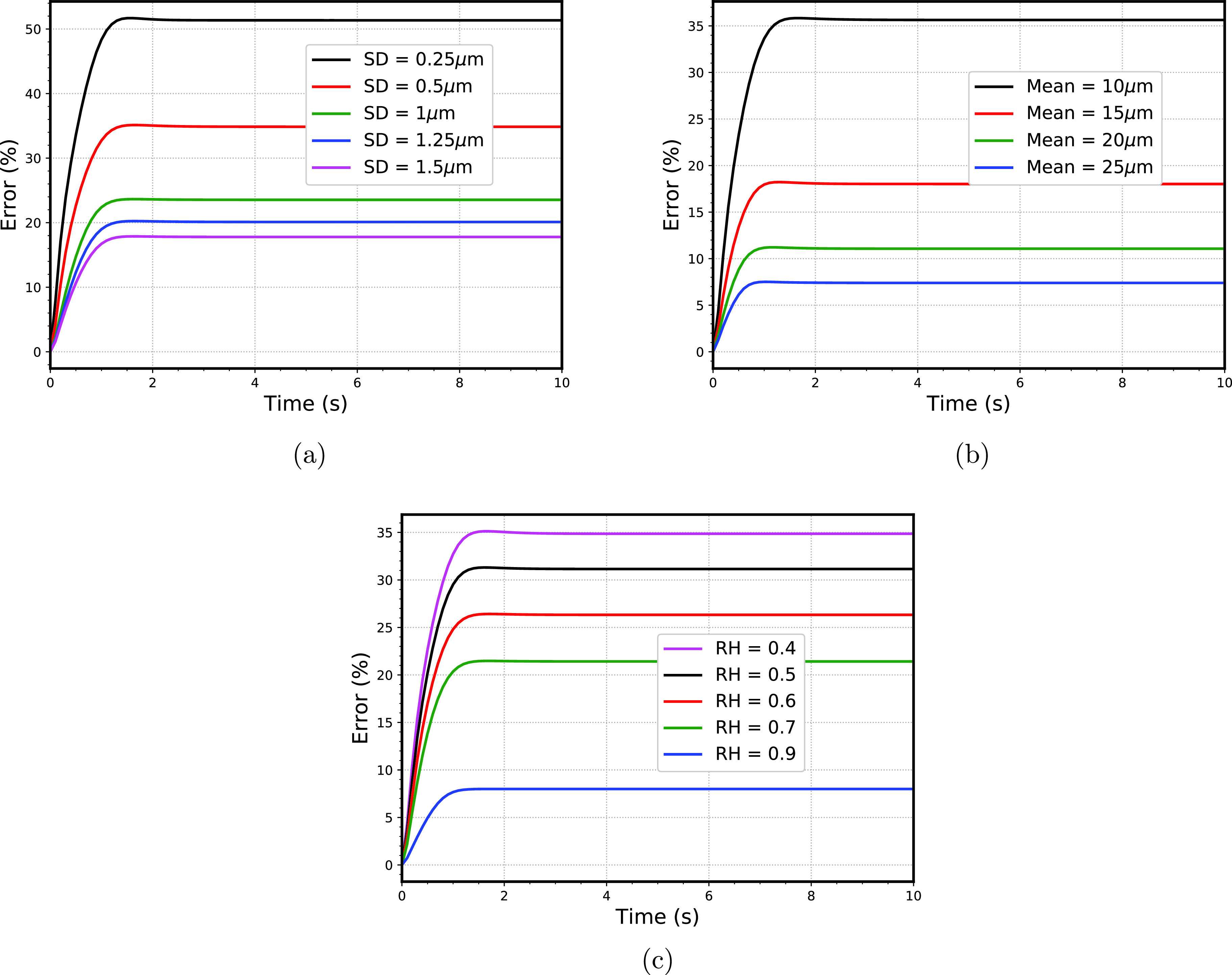
Evolution of error for (a) different initial standard deviation of droplet size spectra (b) different initial mean of droplet size spectra (c) different relative humidity in the sub-saturated section. of the computational domain.

To quantify the difference in the droplet spectra due to various factors and understand their relative effects, we measure the standard *L*^2^ norm error between the size spectra for different cases

$$\text{Error}=\frac{\left||f_{f}-f_{i}|\right|}{\left||f_{i}|\right|},$$ (16)

where, for any size spectra *f*(*r*), the norm is given as $||f||={\left(\int_{0}^{\infty }f{\left(r\right)}^{2}dr\right)}^{1/2}$; $f_{f}(r)$ and $f_{i}(r)$ are the droplet size spectra corresponding to final (time =10 s) and initial (t =0 s), respectively. Figure 10(a) shows the error variation for different initial standard deviation of droplet size spectra. The error is maximum for the initially narrow (standard deviation = 0.25 *μ*m) droplet size spectra and minimum for the broadest (standard deviation = 1.5 *μ*m) droplet size spectra. As pointed out in Sec. V A [Fig. 6(a)], the slope for the narrowest is highest while comparing with other standard deviations. This is not surprising, since now while calculating the error, we are normalizing the final droplet size spectra with respect to the initial droplet size spectra. Also, because the growth rate of the droplets is inversely proportional to the radius of the droplets [refer to Eq. (1)], thus the smaller droplets grow faster as compared to the larger droplets. Figure 10(b) showing the error evolution for different initial mean radius is also not unusual, since as explained in Sec. V B that the presence of larger droplets inhibiting the evaporation of smaller droplets, thereby homogenizing the vapor field in the domain. Consequently, the final droplet size spectra are not extremely different from the initial size spectra. Figure 10(c) shows the effect of relative humidity of the ambient atmosphere on the error, and as expected that with the increase in the humidity contrast, the inhomogeneous mixing leads to the broadening of the droplet size spectra, thereby increasing the probability of the virus-carrying droplet (depending on the severity of the case).

## VI. CONCLUSION

We studied the mixing of coughing/sneezing droplets with the ambient air in the presence of a turbulent velocity field. We varied the droplets characteristics ejected while sneezing/coughing viz. standard deviation and mean radius of the droplet size spectra. We also investigated the effects of the humidity contrast of the ambient atmosphere. The following conclusions can be drawn from this work:

(1) With increasing the standard deviation of the droplet size spectra while maintaining the same mean radius and ambient conditions, the final droplet size spectra broaden. While considering the fact that with increasing the severity of the cases (from mild to severe), the cutoff (virus laden droplets) radius also decreases.^40^ Thus, the broadening of spectra implies that the probability of the virus-laden droplets also increases and thus leads to high transmission.
(2) The presence of larger droplets (or droplet size spectra with higher mean radius) inhibits the broadening of the size spectra of droplets by partially evaporating and providing the necessary vapor to saturate the computational domain.
(3) The environmental humidity contrast also plays a keen role in determining the final droplet size spectra. Increasing the humidity contrast between the sneezing zone and the ambient environment broadens the size spectra of droplets; thus, the drier region has a higher transmission rate. This fact follows the previous works of Refs. 9 and 10. However, it is noted that in this work, we assumed no temperature difference between the droplets and the environment.

## Data Availability

The data that support the findings of this study are available from the corresponding author upon reasonable request.
